# Epidemiology of pneumococcal meningitis in sentinel hospital surveillance of Viet Nam, 2015–2018

**DOI:** 10.1186/s12879-024-10065-0

**Published:** 2024-10-18

**Authors:** Dac Trung Nguyen, Thi Loan Nguyen, Allison Olmsted, Thi Hong Duong, Hong Mai Hoang, Lien Huong Nguyen, Mahamoudou Ouattara, Jennifer Milucky, Fernanda C. Lessa, Thi Trang Dai Vo, Van Thanh Phan, Thi Hien Anh Nguyen, Nguyen My Nguyet Pham, Huu Khanh Truong, Thi Quynh Tram Phan, Thi Hong Hoa Bui, Van Khang Pham, Makiko Iijima, Binh Le, Lindsay Kim, Jennifer Loo Farrar

**Affiliations:** 1 National Institute of Hygiene and Epidemiology, Hanoi, Viet Nam; 2 Centers for Disease Control and Prevention, Atlanta, GA USA; 3 Pasteur Institute in Ho Chi Minh City, Ho Chi Minh, Viet Nam; 4 Ho Chi Minh City Children Hospital No.2, Ho Chi Minh City, Viet Nam; 5 Ho Chi Minh City Children Hospital No.1, Ho Chi Minh City, Viet Nam; 6 National Paediatric Hospital, Hanoi, Vietnam; 7 World Health Organization, Hanoi, Viet Nam; 8 Centers for Disease Control and Prevention, Hanoi, Viet Nam; 9 US Public Health Service, Rockville, MD USA; 10 1600 Clifton Road NE, Atlanta, GA 30329 USA

**Keywords:** Pneumococcus, Meningitis, Children, Asia, Vaccine preventable disease

## Abstract

**Background:**

*Streptococcus pneumoniae (S. pneumoniae)*, *Haemophilus influenzae (H. influenzae)*, and *Neisseria meningitidis* (*N. meningitidis*) are leading causes of childhood bacterial meningitis and preventable by vaccines. The aim of this hospital-based sentinel surveillance is to describe the epidemiological characteristics of pneumococcal meningitis, including disease burden, and to provide baseline data on pneumococcal serotype distribution to support decision making for pneumococcal conjugate vaccine (PCV) introduction in Vietnam.

**Methods:**

Surveillance for probable bacterial meningitis in children 1–59 months of age is conducted in three tertiary level pediatric hospitals: one in Hanoi and two in Ho Chi Minh City. Cerebrospinal fluid (CSF) specimens were collected via lumbar puncture from children with suspected meningitis. Specimens were transferred immediately to the laboratory department of the respective hospital for cytology, biochemistry, and microbiology testing, including culture. PCR testing was conducted on CSF specimens for bacterial detection (*S. pneumoniae*,* H. influenzae*, and *N. meningitidis*) and pneumococcal serotyping.

**Results:**

During 2015–2018, a total of 1,803 children with probable bacterial meningitis were detected; 1,780 had CSF specimens available for testing. Of 245 laboratory-confirmed positive cases, the majority were caused by *S. pneumoniae* (229,93.5%). Of those with *S. pneumoniae* detected, over 70% were caused by serotypes included in currently available PCV products; serotypes 6 A/6B (27.1%), 14 (19.7%), and 23 F (16.2%) were the most common serotypes. Children with laboratory-confirmed pneumococcal meningitis were more likely to live in Hanoi (*p* < 0.0001) and children 12–23 months of age were at greater odds (OR = 1.65, 95% CI: 1.11, 2.43; *p* = 0.006) of having confirmed pneumococcal meningitis compared to children < 12 months of age when compared to those without laboratory-confirmed bacterial meningitis. Additionally, children with confirmed pneumococcal meningitis were more likely to exhibit signs and symptoms consistent with clinical meningitis compared to negative laboratory-confirmed meningitis cases (*p* < 0.0001) and had a greater odds of death (OR = 6.18, 95% CI: 2.98, 12.86; *p* < 0.0001).

**Conclusions:**

Pneumococcal meningitis contributes to a large burden of bacterial meningitis in Vietnamese children. A large proportion are caused by serotypes covered by PCVs currently available. Introduction of PCV into the routine immunization program could reduce the burden of pneumococcal meningitis in Viet Nam.

## Background

Bacterial meningitis can be a great cause for concern in children aged < 5 years due to its severity. It has a high fatality rate, requires urgent medical care and management, and survivors can have severe lifelong disability, significantly burdening families, communities, and societies [1]. A common pathogen that causes bacterial meningitis in children includes *Streptococcus pneumoniae (S. pneumoniae*).

With proven effectiveness of pneumococcal conjugate vaccines (PCV) to reduce not only meningitis, but also pneumonia and other invasive disease burden, the World Health Organization (WHO) has recommended the implementation of this vaccine globally [2]. As of February 2023, 168 Member States had introduced and another 16 were planning to introduce PCV [3]. Recent estimates showed that the recent introduction of PCV in countries with high child mortality is associated with reductions in diseases and deaths caused by *S. pneumoniae*. Globally, pneumococcal-associated death declined 51% from 2000 to 2015 [4].

PCV has not yet been introduced into the Viet Nam EPI but has been available through the private immunization sector since 2013. Viet Nam is planning to introduce PCV in the near future.

Prior to 2009, there was no surveillance for bacterial meningitis, and limited studies have documented the burden of bacterial meningitis disease in Viet Nam [5–9]. A prospective, hospital-based study conducted in Central and Southern Viet Nam in 2007–2010 reported 25% of bacterial meningitis in children less than 15 years old was attributed to *S. pneumoniae* [7]; however, recent data are sparse.

This paper summarizes findings from hospital-based sentinel surveillance for pneumococcal meningitis in Viet Nam from 2015 to 2018, describes characteristics of cases and disease burden, and provides data on pneumococcal serotype distribution to support decision making for PCV introduction.

## Methods

### Description of surveillance system

Prospective active surveillance for probable bacterial meningitis cases (PBM) was conducted at three sentinel hospital sites in Viet Nam: a national pediatric hospital in the North (National Pediatric Hospital in Hanoi) and two provincial pediatric hospitals in the South (Children’s Hospital No. 1 and No. 2 in Ho Chi Minh City). All three pediatric hospitals are large, tertiary referral hospitals likely to treat children presenting with PBM.

### Case definition

For children 1–59 months of age admitted with suspected meningitis (i.e., fever and at least one meningeal sign OR clinical suspicion of meningitis), a lumbar puncture was conducted to collect cerebrospinal fluid (CSF) as the standard of care. Microbiology and biochemistry testing were conducted on CSF specimens, and results were reported back to hospital staff. Hospital staff in the clinical treatment department, laboratory department, and planning department were trained on the surveillance purpose, case definition, and procedure. Children admitted with clinically suspected meningitis as well as at least one of the following CSF findings: (1) turbid appearance, (2) white blood cell (WBC) count > 100 cells/mm^3^, (3) protein > 1 g/L, (4) WBC 10–100 cells/mm^3^ AND protein > 4.5 g/L, or (5) WBC 10–100 cells/mm^3^ AND glucose < 2.2mmol/L (% glucose in CSF / % glucose in blood < 40%) were defined as PBM cases and enrolled in surveillance. Exclusion criteria were (1) cases who met PBM case definition but had an alternative clinical diagnosis, or (2) cases with CSF specimens with visible blood in the tube (if discordance among staff assessing visibility of blood, specimens were excluded using a red blood cell: WBC ratio > 500:1 according to hematology results). A laboratory-confirmed meningitis case was defined as a PBM case with an etiology of *S. pneumoniae*, *Haemophilus influenzae (H. influenzae)*, or *Neisseria meningitidis* (*N. meningitidis)* detected in CSF by real-time polymerase chain reaction (rt-PCR) using laboratory methods previously described [10]. A pneumococcal meningitis case was defined as a laboratory-confirmed meningitis case with *S. pneumoniae* detected in CSF by rt-PCR.

### Data collection

Data on patient demographics, clinical symptoms, vaccination history, and patient outcomes were collected by physicians from the medical record, vaccination cards, and interviews with the person accompanying the child using a standardized case report form. Clinical signs and symptoms were collected during a routine medical examination by a pediatrician. The level of consciousness was evaluated using the Glasgow Coma Scale (GCS) for children 24–59 months old and Pediatric GCS for children < 24 months old [11, 12]. The case report form inquired about use of antibiotics in the week preceding admission and antibiotic name, if applicable. Date of discharge, disease outcomes, and presence of sequelae were collected upon discharge.

### Specimen collection, storage, and transport

CSF specimens were collected among patients identified to have suspected meningitis by clinicians. Collection was done as close to the time of hospital admission as possible and before antibiotic use at the surveillance sites; however, CSF of patients who already received antibiotics before admission were still accepted. Lumbar punctures were performed by experienced personnel under aseptic conditions as part of the standard of care. Specimens were split into three sterile tubes and transferred immediately to the laboratory department of the hospital for cytology, biochemistry, and microbiology testing, including culture. Prior to centrifugation, laboratory staff used aseptic procedures to aliquot 0.5 ml of CSF into a cryotube. Specimens were stored at ≤-20^o^C until specimen transportation. Once CSF findings identified the patients to be PBM cases, aliquots were transferred for molecular testing at one of the national laboratories: National Institute of Hygiene and Epidemiology (NIHE) or Pasteur Institute of Ho Chi Minh City (PIHCMC). Specimens were transported at < 8^o^C soon after PBM case identification, with a goal of transportation within 24 h of specimen collection, but at least within one week.

### Pathogen detection and pneumococcal serotyping

Bacteriology laboratories at NIHE or PIHCMC conducted real-time PCR testing for bacterial detection and serotyping and/or serogrouping of *H. influenzae*,* S. pneumoniae*, and *N. meningitidis* within one week of receiving specimens in accordance with standard methods used in the WHO IB-VPD global surveillance Network [13]. Laboratory-confirmed cases with *lytA* (*S. pneumoniae*-specific gene target) positive specimens were tested for pneumococcal serotype detection as previously described [14]. Briefly, the method consists of seven sequential triplex reactions (21 assays) that identify 37 pneumococcal capsular serotypes as 11 individual serotypes plus 10 small serogroups. Reaction mixtures were prepared in a final volume of 25 µl, including variable volumes of each primer and probe to reach the desired final concentration, 2 µl of CSF as DNA template, 12.5 µl of mastermix (PerfeCTa^®^ MultiPlex qPCR ToughMix, QuantaBio) and PCR-grade water. The thermal profile condition for the qPCR runs was: 1 cycle of 95 °C for 10 min, followed by 40 cycles of 95 °C for 15 s and 60 °C for 1 min. Each run included appropriate positive control DNA and no negative template controls (NTC). A reaction was considered valid if all NTCs were negative, and control DNA were positive. A subset of negative specimens were tested for quality control.

### Data analysis

All epidemiologic and laboratory data were entered into a Microsoft Access database and analyzed using STATA Version 10 (StataCorp. 2007. *Stata Statistical Software: Release 10*. College Station, TX: StataCorp LP). Children who did not meet inclusion criteria or with unknown age were excluded from the analysis. Demographic characteristics, clinical symptoms, and laboratory results were analyzed descriptively. Serotype data were analyzed in aggregate. Statistical differences between laboratory-confirmed pneumococcal meningitis cases and children without laboratory confirmation among children with PBM were assessed using chi-square or Fisher’s exact test, t-test and odds ratios; p-values < 0.05 were considered statistically significant.

### Ethics statement

All data were collected as part of routine surveillance activities; as such, neither guardian consent nor child assent were required. All activities were approved for implementation by the National Institute of Hygiene and Epidemiology in Hanoi, Viet Nam, but did not require ethics approval as activities were part of routine surveillance. This activity was also reviewed by the Institutional Review Board at CDC, deemed not research, and was conducted consistent with applicable federal law and CDC policy (45 C.F.R. part 46.102(l), (2), 21 C.F.R. part 56; 42 U.S.C. § 241(d); 5 U.S.C. § 552a; 44 U.S.C. § 3501 et seq.).

## Results

During January 2015–December 2018, a total of 1,803 children aged 1–59 months met the PBM case definition and were reported across three sentinel hospitals. Of 1,803 PBM cases meeting the case definition, 1,780 (98.7%) had residual CSF specimens available for PCR testing.

**Fig. 1:**
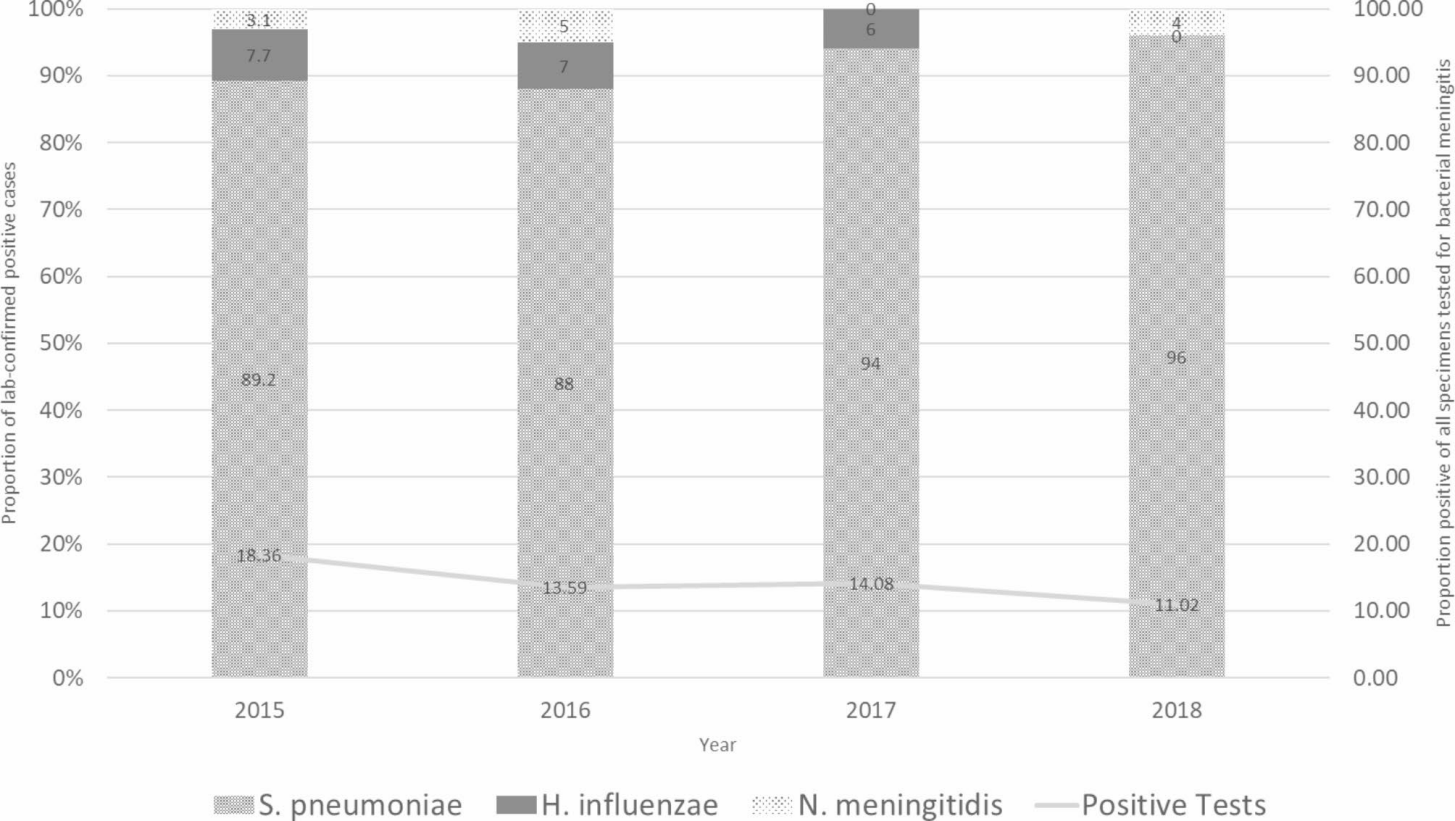
Proportion of laboratory-confirmed positive cases caused by specific pathogens, Viet Nam 2015–2018 (*N* = 1,780)

Among 245 laboratory-confirmed positive cases, 229 (92.7%) were caused by *S. pneumoniae*. Thirteen (5.3%) were *H. influenzae*; of which, 8 (61.5%) were type b, one was type f, and the remaining four were non-typable. Seven (2.9%) cases were caused by *N. meningitidis*, 6 of which were serogroup B and the remaining case was non-typeable. Four cases were positive for both *S. pneumoniae* and *H. influenzae*. The number of specimens tested for bacterial meningitis increased from 2015 to 2018 (Fig. 1). For each year, over 85% of laboratory-confirmed positive cases were caused by *S. pneumoniae*.

The majority of pneumococcal meningitis cases were among children < 12 months of age (162/229, 70.7%), and 60.3% (138/229) were among males (Table 1). All children with pneumococcal meningitis presented with fever; other common symptoms included vomiting (179/229, 78.2%), behavioral change (157/229, 68.6%) convulsions (150/229, 65.5%), and altered consciousness (149/229, 65.1%). Average GCS among pneumococcal-confirmed positive cases was 12.7 with 109/229 (47.6%) between 8 and 12 and 111/229 (48.5%) between 13 and 15. There were 14/229 (6.1%) deaths. Eight children had some history of PCV vaccination, although specific details regarding date of receipt, number of doses, and product received are unknown. Children with pneumococcal meningitis were more likely to live in Hanoi (*p* < 0.0001) and children 12–23 months of age were at greater odds (OR = 1.65, 95% CI: 1.11, 2.43; *p* = 0.006) of having confirmed pneumococcal meningitis compared to children < 12 months of age. Clinically, laboratory- confirmed pneumococcal meningitis cases were more likely to have convulsions, altered consciousness, neck stiffness, behavioral change, vomiting, bulging fontanel, a GCS between 8 and 12 (OR = 4.29, 95% CI: 3.20, 5.77; *p* < 0.0001) and had greater odds of death (OR = 6.18, 95% CI: 2.98, 12.86; *p* < 0.0001) compared to laboratory-confirmed negative meningitis cases (*p* < 0.0001).

**Fig. 2:**
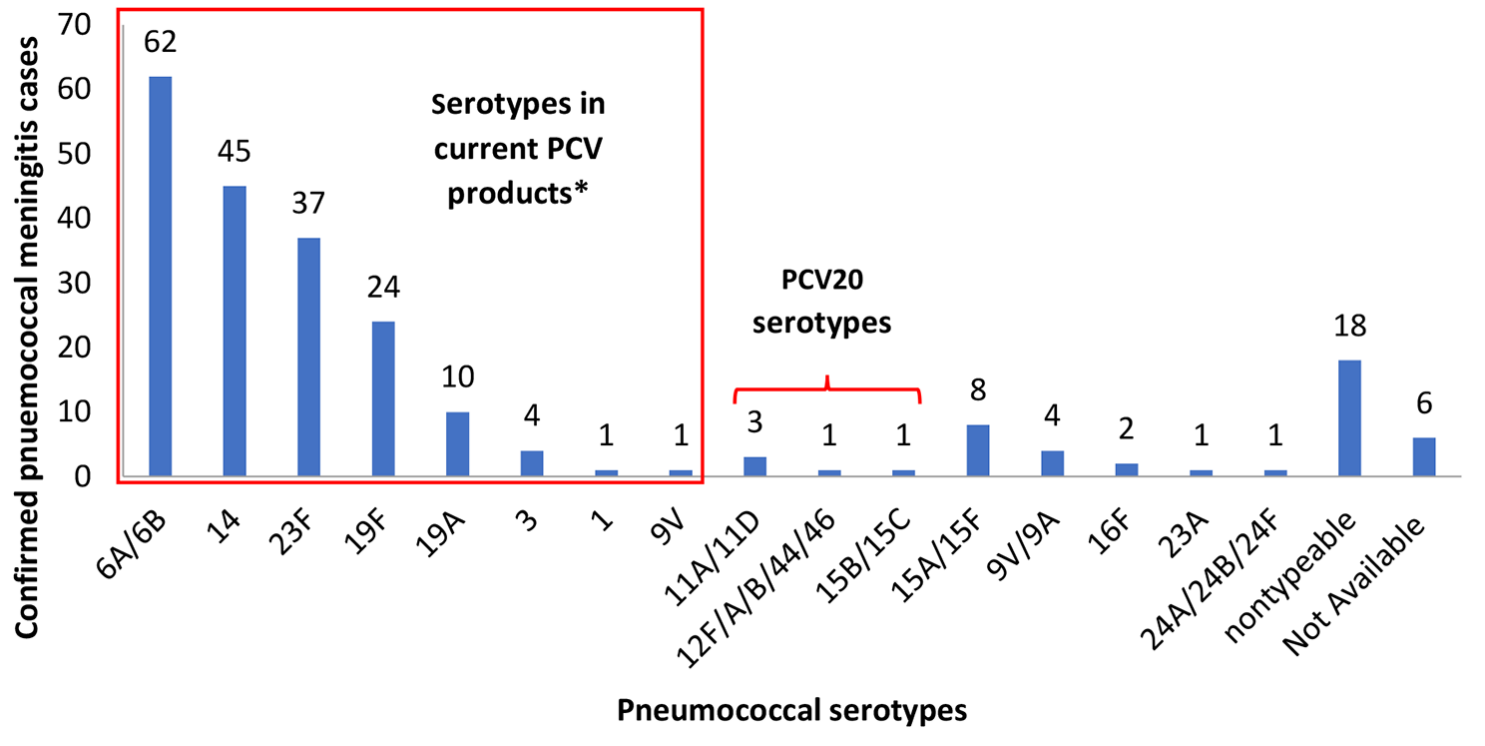
Serotype distribution of *Streptococcus pneumoniae* confirmed cases from surveillance in Viet Nam, 2015–2018 (*N* = 229) Abbreviations: PCV = pneumococcal conjugate vaccine; PCV20 = 20-valent pneumococcal conjugate vaccine *Serotypes in current PCV products includes PCV10SII serotypes (1, 5, 6A, 6B, 7F, 9V, 14, 19A, 19F and 23F), PCV10GSK serotypes (1, 4, 5, 6B, 7F, 9V, 14, 18C, 19F and 23F), and PCV13 serotypes (1, 3, 4, 5, 6A, 6B, 7F, 9V, 14, 18C, 19A, 19F, 23F)

The majority (184/229, 80%) of pneumococcal meningitis cases were caused by serotypes included in currently available PCV products [PCV10 (Synflorix), 74.2%; PCV10 (Pneumosil), 78.6%; PCV13 (Prevnar13), 80.3%]; (Fig. 2).

The most common vaccine-type serotypes were 6A/6B (62/229, 27%), 14 (45/229, 19.7%), and 23F (37/229, 16.2%). Five additional cases were caused by serotypes included in 20-valent PCV (Prevnar20). The remaining pneumococcal meningitis cases were caused by non-vaccine serotypes (16/229, 6.9%) or were non-typable/serotype not available (24/229, 10.5%). Of the non-vaccine serotypes, 15A/15F (*n* = 8) and 9V/9A (*n* = 4) were the most common.

**Table 1:** Demographic and clinical characteristics of probable bacterial meningitis cases in Viet Nam, 2015–2018, by laboratory-confirmation

	Laboratory-confirmed pneumococcal cases *N* = 229 (%)	Laboratory-confirmed negative *n* = 1535 (%)	*p*-value^a^
*Geographic location*			
* Ho Chi Minh City*	76 (33.2)	1045 (68.1)	< 0.0001
* Hanoi*	153 (66.8)	490 (31.9)	
*Age group*			
< 12months	162 (70.7)	1080 (70.4)	0.002
12–23months	38 (16.6)	154 (10.0)	
24–59months	29 (12.7)	301 (19.6)	
*Sex*			
Male	138 (60.3)	988 (64.4)	0.22
Female	91 (39.7)	546 (35.6)	
*Signs and symptoms*			
Fever	229 (100.0)	1515 (98.7)	0.08
Headache	31 (13.5)	268 (17.5)	0.14
Convulsion	150 (65.5)	509 (33.2)	< 0.0001
Altered consciousness	149 (65.1)	363 (23.6)	< 0.0001
Neck stiffness	113 (49.3)	270 (17.6)	< 0.0001
Behavioral change	157 (68.6)	445 (29.0)	< 0.0001
Vomiting	179 (78.2)	849 (55.3)	< 0.0001
Bulging fontanel	74 (32.3)	274 (17.9)	< 0.0001
*GCS* ^*b*^			
Mean (SD)	12.7 (12.4–12.9)	14.1 (14.0–14.2)	< 0.0001
< 8	2 (0.8)	8 (0.5)	< 0.0001
8–12	109 (47.6)	276 (18.0)	
13–15	111 (48.5)	1207 (78.6)	
*CSF results*			
Turbid appearance	142 (62.0)	299 (19.5)	< 0.0001
WBC > 100/mm3	163 (71.2)	994 (64.8)	0.06
Protein > 1 g/l	163 (71.2)	414 (27.0)	< 0.0001
WBC 10–100/mm3 AND Protein > 0.45 g/l	33 (14.4)	446 (29.1)	< 0.0001
WBC 10–100/mm3 AND Glucose < 2.2mmol/l	15 (6.6)	57 (3.7)	0.04
*Outcome*			
Recovered	207 (90.4)	1463 (95.3)	< 0.0001
Died	14 (6.1)	16 (1.0)	
Transferred	1 (0.4)	9 (0.6)	

Abbreviations: PBM = probable bacterial meningitis; GCS = Glasgow Coma Score; CSF = cerebrospinal fluid; WBC = white blood cell

^a^Chi-square test, unless otherwise indicated; t-test used to calculate mean GCS; *p* < 0.05 considered statistically significant. Missing individual symptom and CSF result data were assumed as “no” if not indicated as “yes” due to the nature of data collection

^b^Scores can be interpreted as follows: ≤ 12 suggests severe head injury; < 8 suggests the possibility of intubation and mechanical ventilation

## Discussion

Sentinel site surveillance in Viet Nam identified 1,803 cases of PBM from 2015 thru 2018; 245 were laboratory-confirmed with a bacterial etiology of *H. influenzae*,* N. meningitidis*, or *S. pneumoniae*. We observed regional differences in detection of pneumococcal-confirmed cases between the North (Hanoi) and the South (Ho Chi Minh City). We suspect this could be due to differences in hospital levels; Hanoi Children’s Hospital is a national-level hospital, while Ho Chi Minh City Children’s Hospitals 1 and 2 are administered at the provincial level. Therefore, it is possible that more severe cases are routinely admitted at the Northern site. Most of our laboratory-confirmed meningitis cases were caused by *S. pneumoniae*, over 70% of which were serotypes included in currently available PCV products. The most common serotypes among laboratory-confirmed cases were 6A/6B, 14, and 23F. These findings are consistent with previous studies in Viet Nam and other Asian countries [15, 16], which also observed a predominance of vaccine-type serotypes contributing to pneumococcal disease burden and highlight the need for consideration of PCV introduction into the national immunization program.

With greater PCV implementation into national immunization programs, PCV effectiveness (VE) against invasive pneumococcal disease (IPD), including pneumococcal meningitis, as well as other clinical syndromes caused by *Streptococcus pneumoniae* and reductions in pneumococcal carriage have been demonstrated in children. A systematic review by Berman-Rosa summarized PCV10 (Synflorix) and PCV13 (Prevnar13) effectiveness in children aged < 5 years [17]. Studies using PCV13 on a 3 + 1 schedule reported VE against PCV13-type IPD ranging from 86 to 96% and 67.2–86% for a 2 + 1 schedule. Similarly, studies evaluating PCV10 VE reported 72.8–100% effectiveness against PCV7-type IPD using 3 + 1 schedules and 92–97% using 2 + 1 schedules. Countries in the Asian region have also demonstrated decreased incidence of IPD as well as decreased pneumococcal carriage following national introduction of PCV vaccine [18–20]. These data support that widespread use of PCV in Viet Nam through introduction into the National EPI could mitigate the burden of bacterial meningitis and other clinical outcomes caused by *Streptococcus pneumoniae*.

In Viet Nam, 10-valent PCV (PCV10, Synflorix) manufactured by GSK, and 13-valent PCV (PCV13, Prevnar13), manufactured by Pfizer, have been licensed and available in the domestic market since 2013 (Synflorix) and 2018 (Prevnar13). These vaccines have been utilized mostly among urban children who self-pay. Coverage of these vaccines is unknown but likely limited to small numbers of urban children in several cities such as Hanoi and Ho Chi Minh City in recent years. Competing priorities such as the COVID-19 pandemic and Viet Nam’s graduation from Gavi financing may have further delayed wider PCV implementation. The government of Viet Nam prioritized the introduction of PCV into the national EPI and included PCV introduction in the national workplan, with an anticipated start in the next 1–3 years.

Our surveillance data are subject to a few limitations. First, detected cases likely underestimate the burden of meningitis cases in Viet Nam. The National EPI, WHO, and the U.S. CDC conducted a retrospective review of PBM cases at sentinel sites from 2016 to 2018 and identified cases that were not included in surveillance. Cases were missed due to a lack of clinician sensitization of the surveillance, especially in hospital units that tend to have lower admission rates or treated other diagnoses (e.g., cancer, immunosuppression). Following this review, Viet Nam updated the case definition beginning in 2020 to enroll cases according to the WHO Vaccine-Preventable Diseases Surveillance Standards [21]. Procedures were also modified to improve cross-department collaboration on surveillance within hospitals. Furthermore, the quality of specimen collection, storage, and transport could have led to a lower rate of laboratory-confirmed cases; refresher training to reinforce adherence to standard protocols was conducted to increase quality of specimen collection and transport. Second, data collection was limited to three sentinel sites and may not be representative of the epidemiology of meningitis across the entire country.

## Conclusion

Pneumococcal meningitis contributes to a large burden of laboratory-confirmed bacterial meningitis in Vietnamese children aged < 5 years. Additionally, 70–80% of pneumococcal meningitis cases found during sentinel site surveillance for PBM were caused by serotypes included in currently available PCVs. These surveillance findings suggest that the implementation of PCV into the routine national immunization program could be beneficial to reducing pneumococcal meningitis cases in the future.

## Data Availability

The datasets analyzed during the current study are not publicly available as they are part of disease surveillance.
